# Diversity of Antifungal Properties in Bacterial Isolates from Different Plant Species Growing Across Uzbekistan

**DOI:** 10.3390/microorganisms13051161

**Published:** 2025-05-20

**Authors:** Mukhlisa K. Shodmonova, Dono A. Muhammadova, Ilkham S. Aytenov, Marufbek Z. Isokulov, Tohir A. Bozorov, Daoyuan Zhang, Ozodbek S. Abduraimov, Sojida M. Murodova, Fazliddin A. Melikuziev, Bekhruz O. Ochilov, Sodir K. Meliev

**Affiliations:** 1 Laboratory of Molecular and Biochemical Genetics, Institute of Genetics and Plants Experimental Biology, Uzbek Academy of Sciences, Tashkent Region, Yukori-Yuz, Kibray 111226, Uzbekistan; muxlisashodmonova1994@gmail.com (M.K.S.); donoxyz91@gmail.com (D.A.M.); ilhamaytenov@gmail.com (I.S.A.); isoqulovmarufbek@gmail.com (M.Z.I.); murodovasojida1433@gmail.com (S.M.M.); fmelikuziev23@gmail.com (F.A.M.); behruz.ochilov1995@mail.ru (B.O.O.); meliev.sodir@mail.ru (S.K.M.); 2 Key Laboratory of Ecological Safety and Sustainable Development in Arid Lands, Xinjiang Institute of Ecology and Geography, Chinese Academy of Sciences, Urumqi 830011, China; zhangdy@ms.xjb.ac.cn; 3 Xinjiang Key Laboratory of Conservation and Utilization of Plant Gene Resources, Xinjiang Institute of Ecology and Geography, Chinese Academy of Sciences, Urumqi 830011, China; 4 Laboratory of the Cadastre of Natural Plant Resources and Population Biology, Institute of Botany, Uzbek Academy of Sciences, Tashkent 100125, Uzbekistan; ozodbek88@bk.ru

**Keywords:** antagonistic bacteria, antifungal activity, biocontrol, enzymatic activities, plant pathogens

## Abstract

Plant-associated bacteria play a crucial role in protecting plants from pathogens, yet the diversity and antagonistic potential of these bacteria across different plant species remain underexplored, especially in central Asia. To investigate the competitive dynamics between phytopathogenic fungi and plant-associated bacteria, we collected stem and root samples from 50 plant species across nine regions of Uzbekistan. A total of 3355 bacterial isolates were obtained (1896 from roots and 1459 from shoots) and screened for antifungal activity against six fungal pathogens, resulting in 432 antagonistic isolates. These were identified through 16S rDNA sequencing, revealing 65 bacterial species across three phyla: Firmicutes, Proteobacteria, and Actinobacteria, predominantly in the respective families *Bacillaceae*, *Pseudomonadaceae*, and *Caryophanaceae.* The plant *Salsola vvedenskii* hosted the highest diversity of antagonists (26 species), while other species harbored fewer. Plant species showed strong associations with specific bacterial communities, with 14 plant species each hosting unique antagonists. Enzymatic profiling revealed functional diversity, with *Bacillus* species producing protease, cellulase, and lipase activities, while *Pseudomonas* species excelled in xylanase, glucanase, and cellobiase production. *B. mojavensis* 9r-29 stood out by producing all six enzymes. These findings underscore the ecological diversity and biocontrol potential of plant-associated bacteria in natural ecosystems, offering promising candidates for sustainable plant protection strategies.

## 1. Introduction

Plants are constantly exposed to a wide range of fungal pathogens that can cause significant damage to agricultural crops and can disrupt ecosystems [1]. Fungal pathogens are responsible for diseases that reduce crop quality, diminish yields, and in some cases, lead to complete crop failure. In response to these threats, plants have evolved sophisticated immune systems and established relationships with beneficial microorganisms, including antagonistic bacteria.

Antagonistic bacteria play a crucial role in maintaining plant health and ecological balance by naturally suppressing the growth of harmful phytopathogens [2]. These beneficial microbes, residing in niches such as the rhizosphere (below ground), phyllosphere (above ground), and as endophytes within plant tissues [3], exert their protective effects through various mechanisms such as competition for nutrients and space, production of antimicrobial compounds, including antibiotics, lipopeptides (e.g., surfactin, iturin, and fengycin), and polyketides, and secretion of hydrolytic enzymes that degrade pathogen cell walls [4,5]. These bioactive compounds can inhibit fungal growth by disrupting fungal cell membranes, interfering with key metabolic processes, or blocking spore germination [6]. Unlike chemical fungicides, which often have harmful environmental and health impacts, antagonistic bacteria provide a safer alternative. In agriculture, the use of antagonistic bacteria presents a sustainable alternative to chemical pesticides, helping to reduce environmental pollution and the development of pathogen resistance [7]. Moreover, many of these bacteria promote plant growth by enhancing nutrient uptake, producing phytohormones, and improving soil structure [8]. These bacteria play a crucial role in protecting plants by suppressing fungal pathogens and promoting overall plant health through various biological mechanisms [9,10].

In addition to producing antifungal compounds, antagonistic bacteria also compete with fungal pathogens for nutrients and space, a process known as competitive exclusion. This mechanism is particularly important in controlling soil-borne pathogens, where the availability of resources is essential for pathogen establishment [6]. Furthermore, antagonistic bacteria can enhance plant immunity by triggering systemic resistance (ISR), a phenomenon where the plant’s immune system is primed to respond more effectively to future pathogen attacks. This process leads to enhanced plant resistance not only against fungal pathogens but also against other types of pathogens. For example, *Pseudomonas fluorescens* has been shown to induce ISR in plants, improving their resistance against *Fusarium* and *Phytophthora* species [9]. Additionally, antagonistic bacteria produce lytic enzymes, such as chitinases and glucanases, that break down fungal cell walls, weakening the pathogen and inhibiting its growth. Some bacteria also release volatile organic compounds (VOCs), which act at a distance to suppress fungal growth in both soil and aerial environments [9,11,12].

As concerns about the negative impacts of chemical fungicides grow, antagonistic bacteria have become an attractive and sustainable alternative for managing plant diseases. The use of biocontrol agents, such as antagonistic bacteria, offers several benefits over traditional chemical treatments, including target specificity, environmental safety, and reduced pathogen resistance. Advances in genomic and metagenomic techniques have further expanded the discovery of novel antagonistic bacteria with broad-spectrum activity, opening up new opportunities for biocontrol applications. The incorporation of these bacteria into integrated pest management (IPM) systems can help manage fungal diseases more effectively while reducing reliance on harmful chemical fungicides [13,14,15].

The potential of antagonistic bacteria extends beyond disease control; these bacteria also play an important role in promoting plant growth and enhancing tolerance to biotic and abiotic stresses. The growing interest in sustainable pest management strategies has led to an increased focus on biopesticides. For instance, certain species of *Bacillus*, *Pseudomonas*, and *Streptomyces* not only protect plants from fungal pathogens but also support plant growth by improving nutrient uptake and promoting resilience to environmental stressors [16,17]. The widespread use of *Bacillus* species in biocontrol, with a significant percentage of commercially available biopesticides containing strains of this genus, highlights their importance in sustainable agriculture. Approximately 74% of commercially available biopesticides used to control plant diseases contain *Bacillus* strains. For example, *Bacillus subtilis* has shown efficacy in controlling fungal pathogens, including *Rhizoctonia*, *Rosellinia*, and *Fusarium* species [18]. These advancements underscore the potential of harnessing beneficial microorganisms like antagonistic bacteria to develop more sustainable and eco-friendly pest management strategies, which are essential for maintaining crop health and ensuring long-term agricultural productivity [6,9,19,20].

In this study, we investigated the antagonistic properties of bacteria associated with plants from different regions of Uzbekistan, specifically against various pathogenic fungi. The aim of this study was to identify antagonistic *Bacillus* spp. and *Pseudomonas* spp., along with their characteristics, relative abundance, strain-specific antagonistic metabolites, and their potential for use as biofertilizers. Based on their biocontrol potential, *Bacillus* spp. were identified as effective against phytopathogens affecting key agricultural crops, and their properties described in detail. Our research involves several steps: isolating bacteria from different parts of collected plants, assessing their antagonistic activity against plant pathogens, performing molecular identification of the antagonistic bacteria, and studying the enzymatic activities of these bacteria. This approach contributes to sustainable agriculture by reducing the reliance on chemical fertilizers and limiting the impact of disease-causing pathogens on plant health and productivity through the use of beneficial microorganisms.

## 2. Materials and Methods

### 2.1. Sample Location, Plant Materials, and Fungal Pathogens

Plant samples were collected in 2022 from various ecological environments across different regions of Uzbekistan, including areas affected by drought, salinity, and upland soils. GPS coordinates and elevations were recorded. Uzbekistan has a continental climate, as classified by the Koppen system [21]. This means the country experiences cold winters and long, hot, and dry summers. The southern areas have a hot and dry climate, while the mountainous and sub-mountainous regions receive more rainfall and have relatively cooler temperatures.

Both leaf and root organs were collected, placed in sterile bags, transported to the laboratory, and stored at 4 °C for future use. Several phytopathogenic fungi, including *Rhizoctonia solani*, *Aspergillus terreus*, *Aspergillus flavus*, *Fusarium solani*, *Fusarium graminearum*, and *Fusarium culmorum* were obtained from the “Unique Collections of Phytopathogens and Other Microorganisms” at the Institute of Genetics and Plant Experimental Biology, Academy of Sciences of Uzbekistan.

### 2.2. Isolation and Purification of Bacteria from the Plant

Bacterial isolation was undertaken following our previous study [22]. Briefly, the collected plant samples were homogenized, and 1 mL of sterilized phosphate-buffered saline (PBS) buffer (137 mM NaCl, 2.7 mM KCl, 1 mM Na_2_HPO_4_, and 1.8 mM KH_2_PO_4_; pH 7.4) was added and mixed thoroughly. The solution was serially diluted up to 10^−6^ using the same sterile buffer. Each diluted sample was plated onto nutrient agar (NA) (0.5% peptone, 0.3% beef extract, 1.5% agar, pH 6.8) (Coolaber, Beijing, China) under a laminar flow cabinet. The plates were incubated in a thermostatic chamber at 28 °C for 48 to 96 h, until bacterial colonies were observed. Each serially diluted solution was repeated three times. Pure cultures were obtained by re-growing the samples on NA plates. The bacterial colonies displayed distinct morphological characteristics on the agar, including form, size, margin, and elevation. Each colony differed in its morphological features. The isolation of antagonistic bacteria followed the methodology in our earlier work [22]. The study assessed the inhibition of fungal mycelial growth by antagonistic bacteria by measuring the distance between the edges of bacterial and fungal growth. This was achieved using the following formula described by Alenezi, et al. [23]:

I (%) = (1 − a/b) × 100(1)

In this formula, ‘a’ denotes the distance from the center of the fungal colony to the edge of bacterial growth, while ‘b’ refers to the radius of the fungal colony in the control group.

### 2.3. DNA Extraction and Molecular Identification

PCR, sequencing and analyzing was achieved following [22]. Sequence assembly and analysis were performed using SeqMan software (V. 7.2.1) from the DNASTAR Lasergene package (v7). The bacterial 16S rRNA sequences were compared with those of related bacterial species in GenBank using the BLASTN algorithm, and sequences with high identity were selected. These sequences were then aligned using CLUSTALW (MEGA7). A maximum likelihood (ML) phylogenetic tree was constructed using the neighbor-joining algorithm, based on the Tajima–Nei model, with 5000 bootstrap replicates in MEGA11.

### 2.4. Determination of Enzymatic Activities

Bacterial cellulase activity was evaluated using a carboxymethylcellulose (CMC)-containing medium [24]. Bacterial isolates were cultured on this medium for 96 h, with CMC as the sole carbon source. After incubation, the colonies were rinsed with water, and Congo red solution (0.5%) was applied for 30 min to stain the agar plates until the CMC became dye-bound [25,26]. To fix the coloration, the plates were rinsed with 1 M NaCl for 5 min and then washed with water. As Congo red binds to cellulose, decolorized zones around the colonies indicated microbial cellulose degradation [26].

Proteolytic activity was assessed on an agar plate [27]. Bacterial isolates were cultured on casein agar plates and incubated at 28 °C for 72 h. Protease activity was observed as clear halos around the colonies, indicating the hydrolysis of proteins by the bacteria.

To assess the bacteria’s lipolytic activity, we used 15 mL of nutrient agar. Cool-filtered Tween 85 was added to the pre-cooled medium and mixed thoroughly. Bacteria were cultured at 30 °C for 96 h. Clear halos surrounding the bacterial colonies were observed, indicating lipolytic activity [28].

Antagonistic bacteria were isolated from the tested bacterial samples. The selected isolates were re-inoculated onto the previously mentioned standard media and grown under the described conditions until healthy colonies appeared. Each of the 30 bacterial isolates was transferred to separate Petri dishes. Substrate solutions of glucanase (4-nitrophenyl β-D-glucopyranoside—30.125 mg), xylanase (4-nitrophenyl β-D-xylopyranoside—27.122 mg), and cellobiase (4-nitrophenyl β-D-cellobioside—46.339 mg) were dissolved in 10 mM PBS buffer (pH 5.0) and sprayed onto the colonies (0.3 µM enzyme solution for each cultivated bacteria) until they were completely covered. The plates were incubated at room temperature for 8 h, with monitoring every 15 min. Substrate hydrolysis and the resulting release of 4-nitrophenyl phosphate (4NP) were indicated by a yellow color. Isolates were scored qualitatively as “0” if no color change occurred, and “1”, “2”, or “3” if coloration appeared within 1, 2, or more hours, respectively. The activities of xylanase, cellobiase, and glucanase were determined by assessing the coloration of their respective substrates [25,29].

### 2.5. Data Analysis

A Sankey plot was generated using the online tool SankeyMATIC. Heatmaps were constructed with the SRPlot online platform (bioinformatics.com.cn) (accessed on 10 March 2025). Quantitative analysis of endophytic bacterial counts and species diversity was performed using Microsoft Excel 2019. All experiments were conducted in three independent replicates. Data are presented as mean ± standard deviation (SD). The geographical coordinates (GPS) of plant collection sites were recorded. Antagonistic bacterial strains were isolated and tested against six phytopathogenic fungi. Their inhibitory effects were assessed by measuring the diameter of the inhibition zones, while enzymatic activity was evaluated based on the size of halo zones formed around the bacterial colonies. For molecular analysis, MEGA software (version 11) was used for sequence alignment and cluster analysis.

## 3. Result

### 3.1. Research Site and Plant Collection

**Figure 1:**
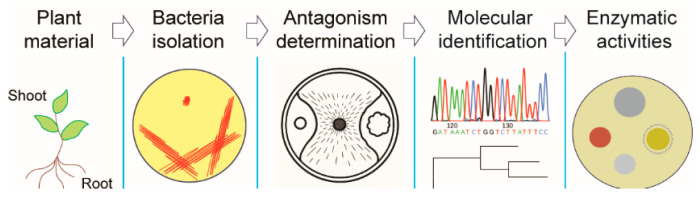
Research strategy for endophytic molecular biodiversity.

To explore the competition between phytopathogenic fungi and plant-associated bacteria, we utilized a range of molecular, microbiological, and analytical techniques. A stepwise approach, based on the method by Aytenov, Bozorov, Zhang, Samadiy, Muhammadova, Isokulov, Murodova, Zakirova, Chinikulov and Sherimbetov [22], was used to identify the bacteria and screen them for antifungal properties against various phytopathogenic fungi, as well as for other relevant activities, as illustrated in Figure 1.

Following this workflow, stem (twig) and root samples were collected from different regions of the country, and pure cultures were isolated from each plant part. Each bacterial isolate was then tested for antifungal activity against six selected fungi. The bacteria that exhibited antagonistic effects were further subjected to molecular identification. Finally, the bacteria showing antifungal activity were assessed for additional enzymatic activities.

**Figure 2:**
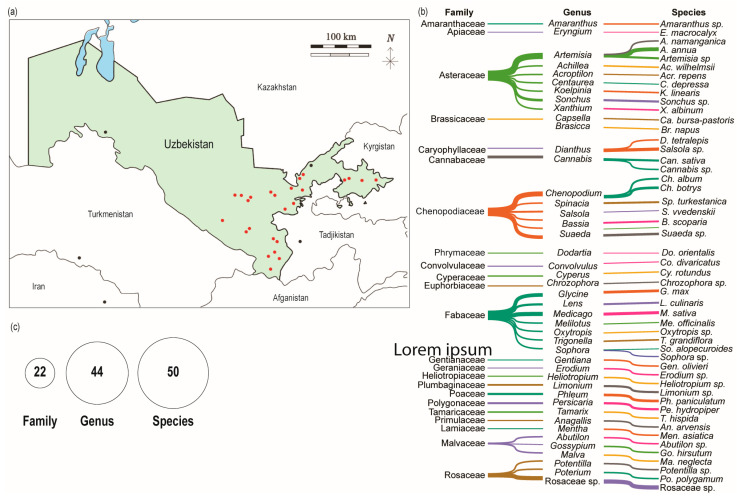
Overview of plant diversity collected from nine regions of Uzbekistan. (**a**) Map of the Republic of Uzbekistan with locations marked for plant sample collection. (**b**) Diversity of plant species categorized by family and genus. (**c**) Distribution of plant species by family and genus.

A total of fifty randomly selected plant species, representing families such as Amaranthaceae, Apiaceae, Asteraceae, Brassicaceae, Caryophyllaceae, Cannabaceae, Chenopodiaceae, Phrymaceae, Convolvulaceae, Cyperaceae, Euphorbiaceae, Fabaceae, Geraniaceae, Gentianaceae, Heliotropiaceae, Plumbaginaceae, Poaceae, Polygonaceae, Tamaricaceae, Primulaceae, Lamiaceae, Malvaceae, and Rosaceae, were collected from nine regions of Uzbekistan: Andijan, Jizzakh, Namangan, Navoiy, Kashkadarya, Samarkand, Surkhandarya, Sirdarya, and Tashkent (Figure 2a,b; Table 1). The elevation of the sampling sites ranged from 241 to 1702 m above sea level, reflecting a diversity of ecological environments (Table 1). The collected species were classified into 21 families and 44 genera, as shown in Figure 2c.

### 3.2. Identification of Cultivable Plant Associated Antagonistic Bacteria

**Figure 3:**
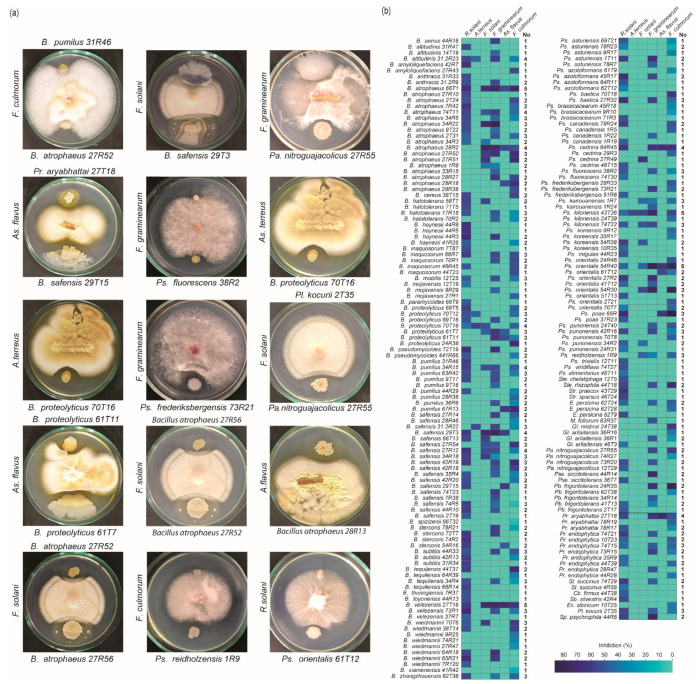
Antifungal Activity of Bacterial Isolates from Plant Species. (**a**) Examples of bacterial isolates exhibiting antifungal activity against various phytopathogenic fungi. (**b**) Heatmap illustrating the antifungal activity of these isolates against six different phytopathogenic fungi. The ‘No’ indicates the number of bacterial isolates capable of inhibiting each pathogenic fungus.

Bacterial colonies were randomly selected from various plant parts. Pure isolates were obtained from both the phyllosphere and rhizosphere of different plant species. In total, 3355 bacterial isolates were obtained, with 1896 derived from roots and 1459 from shoots (Table 2). Using a co-cultivation method, 432 out of the 3355 isolates exhibited antifungal activity against six fungal pathogens in vitro (Figure 3). The antagonistic bacterial isolates were subjected to molecular identification through analysis of the 16S rDNA gene fragment. Sequences obtained from these bacteria were compared against publicly available databases using BLAST 1.4.0 searches in GenBank. Analysis revealed that some antagonistic bacterial isolates were capable of inhibiting up to five different pathogenic fungi (Figure 3b). Among the tested fungi, *R. solani* was the most susceptible to bacterial inhibition, whereas *A. terreus* and *F. solani* were the most tolerant (Figure 3b). The results reveal that the most frequently identified bacterial genera were *Bacillus* and *Pseudomonas*. Several individual strains from these genera were capable of inhibiting up to five fungal pathogens. For instance, *B. atrophaeus* 66T1 and *B. velezensis* 27T16 showed antagonistic activity against five fungal pathogens, while *B. atrophaeus* 28R2 inhibited four. Among *Pseudomonas* species, *Ps. orientalis* 54R40 was effective against five fungal pathogens, and *Ps. cedrina* 64R45 inhibited four.

### 3.3. Diversity of Antagonistic Bacteria

**Figure 4:**
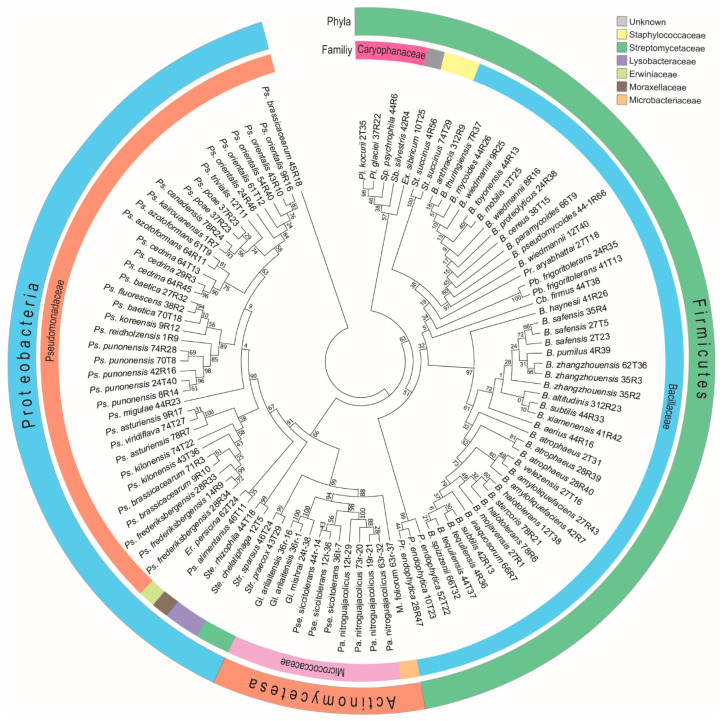
Phylogenic relationship of antagonistic bacteria isolated from plant species. The circular map depicts bacterial phyla and family taxonomic units. The evolutionary history was inferred by using the maximum likelihood method and Tamura–Nei model. The tree with the highest log likelihood (−15,874.16) is shown. The percentage of trees in which the associated taxa clustered together is shown next to the branches.

Out of the 3328 bacterial isolates, only 432 demonstrated antagonistic activity against various pathogenic fungi. Genomic DNA was extracted individually from each of these isolates, and the 16S rRNA gene region was amplified for preliminary identification. Sequencing was performed using the traditional Sanger method. The resulting sequences were analyzed to determine the phylogenetic origin of the endophytic bacteria (Figure 4). A total of 65 bacterial species were identified, spanning three phyla—Firmicutes, Proteobacteria, and Actinobacteria; three classes—Bacilli, Gammaproteobacteria, and Actinomycetia; and seven orders—Micrococcales, Kitasatosporales, Bacillales, Enterobacterales, Lysobacterales, Moraxellales, and Pseudomonadales. These were further grouped into ten families (Microbacteriaceae, Micrococcaceae, Streptomycetaceae, Bacillales, Caryophanaceae, Staphylococcaceae, Erwiniaceae, Lysobacteraceae, Moraxellaceae, and Pseudomonadaceae. In total, 18 genera were represented among the antagonistic isolates, including *Bacillus*, *Cytobacillus*, *Erwinia*, *Exiguobacterium*, *Glutamicibacter*, *Microbacterium*, *Paenarthrobacter*, *Peribacillus*, *Planococcus*, *Priestia*, *Pseudarthrobacter*, *Pseudomonas*, *Psychrobacter*, *Solibacillus*, *Sporosarcina*, *Staphylococcus*, *Stenotrophomonas*, and *Streptomyces* (Table S1).

Phylogenetic analysis of the sequenced bacterial species demonstrated that the three largest clades that were most dominant were Firmicutes followed by Proteobacteria and Actinomycetes. The Bacillaceae family was dominant, followed by Pseudomonadaceae, Caryophanaceae, Micrococcaceae, Streptomycetaceae, Staphylococcaceae, Lysobacteraceae, Microbacteriaceae, Erwiniaceae, Moraxellaceae, and an unknown family (Figure 4).

**Figure 5:**
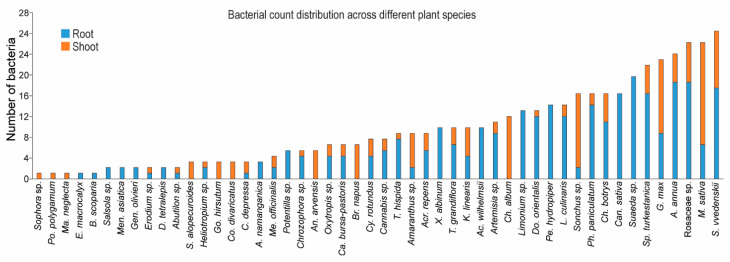
Bacterial count distribution by plant species.

The analysis of plant sources revealed that the majority of antagonistic bacterial isolates are associated with *S. vvedenskii*, which harbored 26 bacteria antagonists. This was followed by *M. sativa* (25), Rosaceae sp. (24), *A. annua* (22), *G. max* (21), *Sp. turkestanica* (20), *Suaeda* sp. (18), *Can. sativa* (15), *Ch. botrys* (15), *Pa. paniculatum* (15), *Sonchus* sp. (15), *L. culinaris* (13), *Pe. hydropiper* (13), *Do. orientalis* (12), *Limonium* sp. (12), *Ch. album* (11), *Artemisia* sp. (10), *Ac. wilhelmsii* (9), *K. linearis* (9), *T. grandiflora* (9), *X. albinum* (9), *Acr. repens* (8), *Amaranthus* sp. (8), *T. hispida* (8), *Cannabis* sp. (7), *Cy. rotundus* (7), *Br. napus* (6), Ca. *bursa-pastoris* (6), *Oxytropis* sp. (6), *An. arvensis* (5), *Chrozophora* sp. (5), *Potentilla* sp. (5), *Me. officinalis* (4), *A. namanganica* (3), *C. depressa* (3), *Co. divaricatus* (3), *Go. hirsutum* (3), *Heliotropium* sp. (3), *S. alopecuroides* (3), *Abutilon* sp. (2), *D. tetralepis* (2), *Erodium* sp. (2), *Gen. olivieri* (2), *Men. asiatica* (2), *Salsola* sp. (2), *B. scoparia* (1), *E. macrocalyx* (1), *Po. polygamum* (1), and *Sophora* sp. (1) (Figure 5). It appears that the majority of antagonistic bacteria were found in the roots compared with the shoots. Antagonistic bacteria were detected in the shoots of only nine plant species, whereas thirteen were identified in the roots (Figure 5).

**Figure 6:**
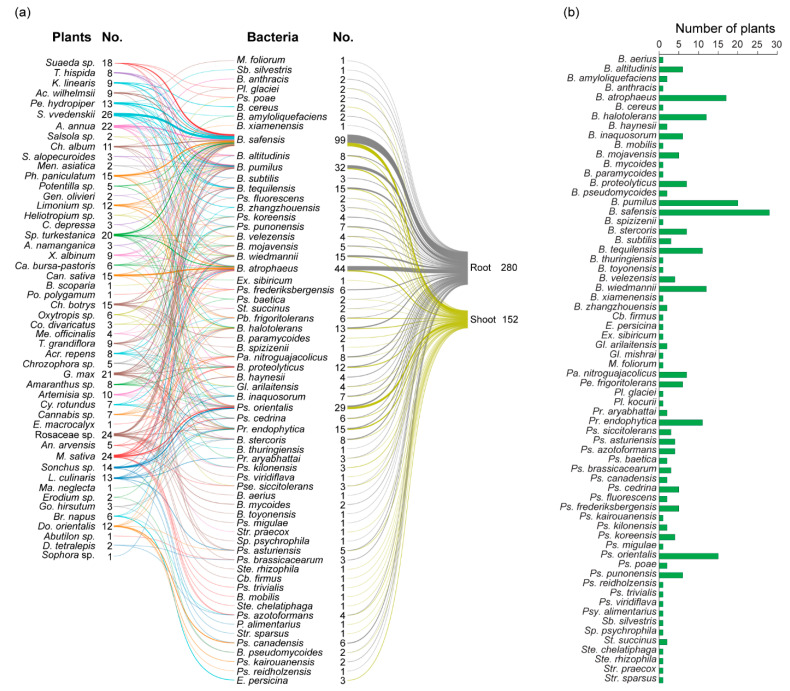
Distribution of antagonistic bacteria by plant species and plant parts. (**a**) The distribution of antagonistic bacterial isolates was analyzed according to the plant species and specific plant parts (roots, shoot) from which they were obtained. (**b**) The data show the distribution of bacterial antagonists based on the number of plant hosts.

Analysis of the distribution across plant organs revealed 280 isolates from the roots and 152 from the shoots, indicating that the roots harbor a higher abundance of antagonistic bacteria. Among the antagonistic bacteria, 21 species—including *Bacillus aerius*, *B. amyloliquefaciens*, *B. anthracis*, *B. haynesii*, *B. mycoides*, *B. subtilis*, *B. thuringiensis*, *B. toyonensis*, *B. xiamenensis*, *M. foliorum*, *Pl. glaciei*, *Ps. brassicacearum*, *Ps. canadensis*, *Ps. frederiksbergensis*, *Ps. kairouanensis*, *Ps. koreensis*, *Ps. migulae*, *Ps. poae*, *Ps. reidholzensis*, *Sp. silvestris*, and *Sp. psychrophila*—were exclusively found in roots. In contrast, 17 species—including *Bacillus cereus*, *B. mobilis*, *B. paramycoides*, *B. spizizenii*, *Cohnella firmus*, *Erwinia persicina*, *Ex. sibiricum*, *Gl. mishrai*, *Pl. kocurii*, *Ps. kilonensis*, *Ps. trivialis*, *Ps. viridiflava*, *Psy. alimentarius*, *Ste. chelatiphaga*, *Ste. rhizophila*, *Str. praecox*, and *Str. sparsus*—were identified in shoots (Figure 6a). Apparently, the highest number of antagonistic bacteria (26 in total) was found in *S. vvedenskii*, which harbored nine different bacterial species. In contrast, the lowest number—only one bacterial species—was observed in *B. scoparia*, *E. macrocalyx*, *M. neglecta*, *Po. polygamum*, and *Sophora* sp. (Figure 6a,b). *B. safensis* was the most widely distributed bacterial species, found in association with 27 different plant species (Figure 6b). Fourteen plant species each harbored distinct bacterial species not found in others (Figure 6a). Most bacterial species, according to plant family, also appeared to be distinct. For example, *Ps. migulae*, *Ste. rhizophila*, *B. toyonensis*, *Cb. firmus*, *B. mycoides*, *B. haynesii*, and *B. aerius* were found to be associated with Rosaceae species. *B. mobilis*, *Ps. trivialis*, *Ste. chelatiphaga*, *E. persicina*, and *Ps. viridiflava* were found in Fabaceae. *Pl. kocurii*, *Pl. glaciei*, *B. paramycoides*, and *B. spizizenii* were detected in Chenopodiaceae, while *B. anthracis*, *B. cereus*, *Gl. mishrai*, *Str. praecox*, *Ps. kairouanensis*, and *Ps. reidholzensis* were found to be associated with Phrymaceae. In Asteraceae, *M. foliorum* and *Str. sparsus* were identified. Additionally, *B. thuringiensis* was specific to Cyperaceae, and *Ex. sibiricum* to Brassicaceae.

### 3.4. Determination of the Enzymatic Activities of the Antagonistic Bacteria

**Figure 7:**
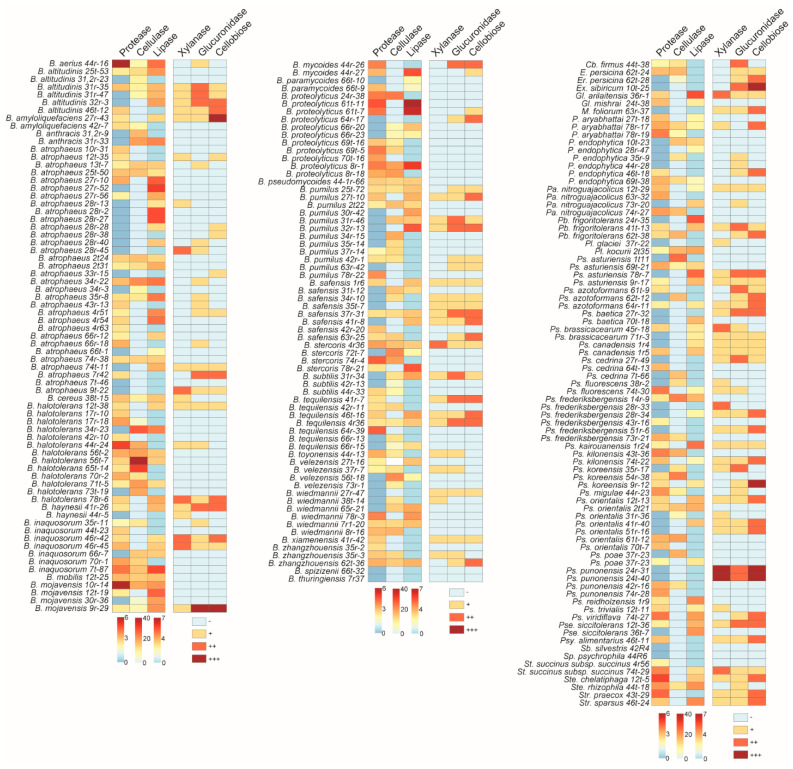
Enzymatic activities of the antagonistic bacteria. Protease, lipase, cellulase, xylanase, glucanase, and cellobiase activities were assessed in antagonistic bacterial isolates. Symbols ‘+’, ‘++’, and ‘+++’ indicate low, moderate, and high levels of enzymatic activity, respectively, while ‘−’ denotes the absence of activity.

The enzymatic properties of antagonistic bacteria isolated from plant roots and shoots were determined, with a focus on their ability to break down cell wall components, as well as degrade lipids and proteins. Among the bacterial genera, *Bacillus* species exhibited the strongest protease, cellulase, and lipase activities, surpassing other genera in these enzymatic functions. Interestingly, the *Bacillus* strains demonstrated variability in their enzymatic degradation capabilities. For instance, *B. mojavensis* 10r-14 displayed the highest protease activity among the strains tested, highlighting its potential as a robust proteolytic agent. On the other hand, *B. halotolerans* 56t-7 showed significant cellulase activity, suggesting its ability to break down complex polysaccharides, while *B. atrophaeus* 27r-52 was particularly effective in lipase activity, which may aid in lipid degradation (Figure 7).

In contrast, *Pseudomonas* species were more effective in exhibiting xylanase, glucanase, and cellobiase activities compared with other bacterial genera. This indicates their potential in breaking down lignocellulosic materials, an important characteristic for bioremediation and plant protection. Similar to *Bacillus* species, *Pseudomonas* strains also exhibited varying levels of enzymatic activity, indicating differences in their metabolic capabilities. For example, *Ps. punonensis* 24r-31 and *Ps. punonensis* 24t-40 showed particularly strong enzymatic activity across multiple enzymes, suggesting a broad range of degradative functions. Additionally, *Ps. koreensis* 9r-12 demonstrated superior cellobiase activity when compared with other *Pseudomonas* strains, highlighting its specific role in breaking down cellulose (Figure 7).

Among all the antagonistic bacteria tested, *B. mojavensis* 9r-29 stood out by demonstrating all six enzymatic activities—protease, cellulase, lipase, xylanase, glucanase, and cellobiase. This versatile enzyme profile positions *B. mojavensis* 9r-29 as a particularly promising candidate for further studies in biocontrol and agricultural applications, where multiple forms of enzymatic degradation are beneficial for plant protection and growth promotion.

## 4. Discussion

The ecological benefits of antagonistic bacteria extend beyond crop productivity, as they contribute to biodiversity, soil fertility, and the resilience of agroecosystems against climate stress and disease outbreaks. As such, antagonistic bacteria represent a valuable resource for developing eco-friendly strategies in modern agriculture and environmental management. The diversity of plant species sampled across nine regions in Uzbekistan, from 241 to 1702 m above sea level, highlights the vast ecological variation within the country. A wide range of plant families were represented, including Amaranthaceae, Apiaceae, Asteraceae, and many others, which emphasizes the extensive potential sources for isolating plant-associated bacteria. The distribution of bacterial isolates from both the phyllosphere and rhizosphere further underlines the significance of plant-associated microorganisms in diverse ecological niches. The finding that roots generally harbor a higher abundance of antagonistic bacteria is consistent with previous research, which suggests that the rhizosphere provides a more nutrient-rich environment conducive to microbial growth and activity [30]. Furthermore, the study also highlights the ecological and phylogenetic diversity of antagonistic bacteria across different plant species and families. Of the 50 identified antagonistic bacterial strains, 37 were found to be associated with more than two plant hosts. This suggests that many bacterial species across the region are capable of colonizing multiple plant types simultaneously. In contrast, some strains exhibited host specificity. For instance, certain bacterial species were found to be specific to particular plant families, such as *Ps. migulae* and *B. aerius*, which were found to be associated with Rosaceae species. This observation suggests that plant species and their associated bacteria may have co-evolved, with certain bacterial strains becoming highly specialized for specific plant hosts. This specialized association between plant species and their microbial communities could have important implications for understanding the dynamics of plant–microbe interactions and for developing targeted biocontrol strategies.

In total, 432 bacterial isolates exhibited antifungal activity against six phytopathogenic fungi, with some bacteria showing the ability to inhibit up to five different fungi. Notably, *R. solani* was the most susceptible to bacterial inhibition, while *As. terreus* and *F. solani* showed greater tolerance. This differential inhibition suggests that certain bacterial strains possess a broader spectrum of antifungal activity, which could be exploited for developing biocontrol agents. The molecular identification of the antagonistic isolates revealed a rich diversity of bacterial species, including those from the Firmicutes, Proteobacteria, and Actinobacteria phyla, with *Bacillus*, *Pseudomonas*, and *Streptomyces* being the most commonly encountered genera. The dominance of *Bacillus* species in this study is particularly noteworthy, as these bacteria are well-known for their antagonistic properties against plant pathogens [22,31,32,33,34]. The wide range of antibacterial compounds synthesized by *Bacillus* species makes them excellent candidates for use as biological control agents (BCAs) in agriculture. Their key antimicrobial metabolites include lipopeptides, siderophores, non-ribosomally synthesized peptides (NRSPs), polyketides, and bacteriocins [6]. Among the *Bacillus* species tested, *B. atrophaeus* exhibited the strongest antifungal activity against most fungal pathogens, followed by *B. safensis*, *B. velezensis*, *B. pumilus*, and *B. proteolyticus*, with most of these producing antifungal compounds such as iturin A, safencin E, cyclo-(D-phenylalanyl-D-prolyl), fengycin, surfactin, bacillaene, macrolactin, difficidin, bacilysin and siderophores (bacillibactin) [35,36,37]. These *Bacillus* strains have been previously reported as promising biological control agents [38,39,40,41,42]. Notably, the *B. atrophaeus* 66T1 strain demonstrated strong inhibitory activity against several *Fusarium* pathogens, including *F. graminearum*, *F. solani*, and *F. culmorum*, consistent with previous studies [43,44]. Guo et al. demonstrated the efficient inhibition of *F. pseudograminearum*, which causes crown rot in wheat, with the disease severity index reduced 76% in a greenhouse pot trial. Since then, the *Fusarium* species is considered to be composed of destructive pathogens in agriculture, and eliminating this pathogen by biological control agents rather than via chemical fungicides is ecologically friendly. *Pseudomonas* species were the second most frequently identified antagonistic microbes associated with plants. For instance, *Ps. orientalis* strain 54R40 exhibited antifungal activity, effectively inhibiting five different fungal pathogens. Another strain, *P. orientalis* F9, is known for its antibacterial properties against *Er. amylovora*, the causative agent of fire blight in apples. This strain produces a range of antibiotics, including safracin and phenazine. Notably, phenazine and its acidic or amidated derivatives are recognized for their potent antifungal activity [29,34,45,46,47]. Another species, *Ps. cedrina* strain 64R45, also exhibited strong antifungal activity, though with a distinct inhibition spectrum. According to Goryluk-Salmonowicz, et al. [48], *P. cedrina* demonstrated significant antifungal effects among thirteen medicinal plant species. The same study also identified other potent antifungal endophytes, including *P. azotoformans*, *B. subtilis*, and *E. persicina*, all of which are referenced in the current study.

One of the most interesting findings of this study was the variation in the enzymatic activities of the antagonistic bacteria. *Bacillus* species exhibited strong protease, cellulase, and lipase activities, surpassing other genera in these enzymatic functions. These activities are essential for breaking down complex macromolecules in the plant cell wall, lipids, and proteins, making *Bacillus* species particularly effective in both biocontrol and plant growth promotion [49,50,51]. Furthermore, certain strains of *Bacillus* demonstrated variability in their enzymatic degradation capabilities, suggesting that different strains might be specialized for distinct types of macromolecular degradation. It has been reported to be an enzymatically diverse bacteria [52,53]. *Pseudomonas* species, on the other hand, are particularly proficient at breaking down lignocellulosic materials, with notable xylanase, glucanase, and cellobiase activities. This ability positions *Pseudomonas* strains as valuable candidates for bioremediation and sustainable agricultural practices.

In conclusion, our study provides valuable insights into the diversity and antifungal potential of plant-associated bacteria. The findings suggest that these bacteria, particularly from genera such as *Bacillus* (*B. atrophaeus*, *B. safensis*, *B. velezensis*, *B. pumilus*, and *B. proteolyticus*) and *Pseudomonas* (*Ps. sedrina*, *Ps. kilonensis*, and *Ps. orientalis*), hold significant promise for biological control applications. Their diverse enzymatic profiles further enhance their potential in various agricultural and bioremediation practices. The discovery of the *B. mojavensis* 9r-29 strain as a particularly versatile bacterium capable of demonstrating all six tested enzymatic activities—protease, cellulase, lipase, xylanase, glucanase, and cellobiase—makes it an exceptional candidate for future bioremediation practices. The broad enzymatic profile of this strain suggests that it could play a significant role in degrading a wide range of plant pathogens and promoting plant health, making it a promising candidate for agricultural applications. Future studies should focus on the in-depth exploration of the mechanisms of action of these bacteria, as well as on their interactions with phytopathogens and their efficacy in field conditions.

## Figures and Tables

**Table 1:** Collection information for plant samples in Uzbekistan.

Location		GPS Coordinates	Elevation (m)	Plant Species
Region	District			
Andijan	Andijan Ulugnor	40°86’53.0 N 72°29′524 E 40°85′15.8 N 71°64′840 E	455 408	*Lens culinaris* *Glycine max*
Jizzakh	Zomin-1 Zomin-2 Zomin-3 Zomin-4 Zomin-5 Rovot-1 Rovot-2 Rovot-3	39°70’58.1 N 68°44’40.0 E 39°70’58.1 N 68°44’39.2 E 39°70’58.1 N 68°44’39.1 E 39°70’58.1 N 68°44’39.0 E 39°70’58.1 N 68°44’38.9 E 40°048’8.1 N 67°58’73.3 E 39°70’58.1 N 68°44’37.1 E 40°04’88.1 N 67°58’72.3 E	1477 1477 1477 1477 1477 241 1477 241	*Eryngium macrocalyx* *Potentilla* sp. *Gentiana olivieri* *Poterium polygamum* *Capsella bursa-pastoris* *Medicago sativa* *Trigonella grandiflora*
Namangan	Pop-1 Pop-2	40°85′61.0 N 70°95′97.2 E 40°91′56.5 N 71°15′44.5 E	503 494	*Chenopodium botrys*, *Heliotropium* sp., *Artemisa namanganica*, *Abutilon* sp., *Amaranthus* sp., *Cannabis* sp., *Glycine max*, *Gossypium hirsutum*
Navoiy	Xatirchi Paxtachi	40°08′09.1 N 65°89′14.3 E 40°09′56.6 N 65°65′80 E	419 372	*Mentha asiatica*, *Artemisia annua* *Tamarix hispida*, *Limonium* sp., *Bassia scoparia*
Kashkadaria	Qamashi-1 Qamashi-2 Qamashi-3 Muborak	38°91’17.9 N 66°66’67.0 E 38°90’738 N 66°66’20.5 E 38°91’57.9 N 66°67’21.2 E 39°30’88.7 N 65°12’01.6 E	600 598 596 284	*Achillea wilhelmsii* *Anagallis arvensis*, *Brasicca napus* *Medicago sativa* *Koelpinia linearis*, *Oxytropis* sp., *Salsola vvedenskii*, *Suaeda* sp.
Samarkand	Nurobod Nurbuloq	39°77′00.2 N 66°42′03.3 E 39°75′39.0 N 66°40′26.8 E	567 543	*Cannabis sativa*, *Centaurea depressa*, *Pseudosophora alopecuroides*, *Spinacia turkestanica* *Acroptilon repens*
Surkhandaria	Khonguzar Sangardak Denov Oltin-Soy Shorchi Jarkurgan Angor	38°60’77.9 N 67°57’12.9 E 38°51’07.9 N 67°65’21.2 E 38°21’57.2 N 67°54’76.4 E 38°048’35 N 67°37’79.9 E 38°03′87.9 N 67°82′80.5 E 37°49′77.5 N 67°42′03.7 E 37°51′52.0 N 67°37′60.7 E	1702 996 793 538 783 348 361	*Dianthus tetralepis*, *Erodium* sp., *Rosaceae* sp. *Sonchus* sp., *Artemisia* sp. *Persicaria hydropiper* *Phleum paniculatum* *Melilotus officinalis* *Malva neglecta* *Chrozophora* sp., *Convolvulus divaricatus*
Syrdaria	Boyovut Sardoba Ok-Oltin Syrdaria	40°43’75.4 N 68°87’12.1 E 40°43’75.4 N 68°87’11.8 E 40°55’47.8 N 68°39’43.0 E 40°90’30.9 N 68°69’39.6 E	279 279 266 253	*Cyperus rotundus* *Salsola* sp. *Artemisia annua* *Xanthium albinum*
Tashkent	Yangiyo’l	41°16′65.4 N 69°05′32.5 E	361	*Chenopodium album*, *Dodartia orientalis*

**Table 2:** Distribution of isolates bacteria by plant species.

Plant Species	Number of Bacterial Isolates		Plant Species	Number of Bacterial Isolates	
	Root	Shoot		Root	Shoot
*Abutilon* sp.	24	24	*Go. hirsutum*	24	24
*Ac. wilhelmsii*	48	21	*Heliotropium* sp.	24	24
*Acr. repens*	48	72	*K. linearis*	24	24
*Amaranthus* sp.	24	36	*L. culinaris*	24	24
*An. arvensis*	24	22	*Limonium* sp.	24	19
*A. namanganica*	48	18	*Ma. neglecta*	48	24
*A. annua*	48	15	*M. sativa*	24	48
*Artemisia* sp.	48	48	*Me. officinalis*	22	24
*B. scoparia*	36	24	*Men. asiatica*	48	25
*Br. napus*	48	48	*Oxytropis* sp.	24	24
*Can. sativa*	46	24	*Pe. hydropiper*	48	24
*Cannabis* sp.	24	24	*Ph. paniculatum*	48	24
Ca. *bursa-pastoris*	36	48	*Potentilla* sp.	62	24
*C. depressa*	24	24	*Po. polygamum*	24	41
*Ch. album*	24	38	*So. alopecuroides*	36	48
*Ch. botrys*	24	36	*Rosaceae* sp.	48	48
*Chrozophora* sp.	48	12	*Salsola* sp.	92	48
*Co. divaricatus*	24	24	*S. vvedenskii*	24	17
*Cy. rotundus*	22	44	*Sonchus* sp.	48	24
*D. tetralepis*	36	13	*Sp. turkestanica*	56	24
*Do. orientalis*	48	24	*Suaeda* sp.	48	24
*Erodium* sp.	72	24	*Ta. hispida*	24	24
*E. macrocalyx*	24	24	*T. grandiflora*	33	24
*Gen. olivieri*	24	24	*X. albinum*	72	46
*G. max*	48	48			
Total	920	759	Total	949	700

## Data Availability

The authors declare that the experimental data published in this paper. will be made accessible upon request for interested readers. All 16S rRNA gene sequences of the new strains can be found under accession numbers: starting from PV653521 to PV653563; and PV653596 to PV653651.

## Supplementary Materials

The following supporting information can be downloaded at: https://www.mdpi.com/article/10.3390/microorganisms13051161/s1, Table S1: Identified bacterial isolates from plant species by 16S RNA sequence analysis and their taxonomic status.
