# Supplementary material for: Diversity of Antifungal Properties in Bacterial Isolates from Different Plant Species Growing Across Uzbekistan
Source: Microorganisms. 2025 May 20;13(5):1161. doi: 10.3390/microorganisms13051161 (PMC12114544; doi:10.3390/microorganisms13051161)
Supplement: Supplementary file 1 [file microorganisms-13-01161-s001.zip › microorganisms-3628633-supplementary.pdf]

**Table S1**. Identified bacterial isolates from plant species by 16S RNA sequence analysis and their taxonomic status.

| Phyum | Class | Order | Family | Species | GenBank ID | Number of isolates |
|---|---|---|---|---|---|---|
| Actinobacteria | Actinomycetes | Micrococcales | Microbacteriaceae | *Microbacterium foliorum* | NR_025368.1 | 1 |
| . | . | | Micrococcaceae | *Glutamicibacter arilaitensis* | NR_074608.1 | 3 |
| . | . | | | *Glutamicibacter mishrai* | NR_169398.1 | 1 |
| . | . | | | *Paenarthrobacter nitroguajacolicus* | NR_027199.1 | 8 |
| . | . | | | *Pseudarthrobacter siccitolerans* | NR_108849.1 | 3 |
| . | . | Kitasatosporales | Streptomycetaceae | *Streptomyces praecox* | NR_115437.1 | 1 |
| . | . | | | *Streptomyces sparsus* | NR_114938.1 | 1 |
| Firmicutes | Bacilli | Bacillales | Bacillaceae | *Bacillus aerius* | NR_118439.1 | 1 |
| | | | | *Bacillus altitudinis* | NR_042337.1 | 8 |
| | | | | *Bacillus amyloliquefaciens* | NR_041455.1 | 2 |
| | | | | *Bacillus anthracis* | MN330338.1 | 2 |
| | | | | *Bacillus atrophaeus* | NR_024689.1 | 44 |
| | | | | *Bacillus cereus* | NR_074540.1 | 2 |
| | | | | *Bacillus halotolerans* | NR_115063.1 | 14 |
| | | | | *Bacillus haynesii* | NR_157609.1 | 4 |
| | | | | *Bacillus inaquosorum* | NR_104873.1 | 7 |
| | | | | *Bacillus mobilis* | NR_157731.1 | 1 |
| | | | | *Bacillus mojavensis* | NR_024693.1 | 5 |
| | | | | *Bacillus mycoides* | MN710449.1 | 2 |
| | | | | *Bacillus paramycoides* | NR_157734.1 | 2 |
| | | | | *Bacillus proteolyticus* | NR_157735.1 | 12 |
| | | | | *Bacillus pseudomycoides* | NR_113991.1 | 2 |
| | | | | *Bacillus pumilus* | NR_043242.1 | 32 |
| | | | | *Bacillus safensis* | NR_113945.1 | 99 |
| | | | | *Bacillus spizizenii* | NR_112686.1 | 1 |
| | | | | *Bacillus stercoris* | MW828060.1 | 8 |
| | | | | *Bacillus subtilis* | MT509759.1 | 3 |
| | | | | *Bacillus tequilensis* | NR_104919.1 | 15 |
| | | | | *Bacillus thuringiensis* | NR_043403.1 | 1 |
| | | | | *Bacillus toyonensis* | NR_121761.1 | 1 |
| | | | | *Bacillus velezensis* | NR_075005.2 | 4 |
| | | | | *Bacillus wiedmannii* | NR_152692.1 | 15 |
| | | | | *Bacillus xiamenensis* | NR_148244.1 | 1 |
| | | | | *Bacillus zhangzhouensis* | NR_148786.1 | 3 |
| | | | | *Cytobacillus firmus* | NR_112635.1 | 1 |
| . | | | | *Peribacillus frigoritolerans* | NR_115064.1 | 6 |

| | | | | | | |
|---|---|---|---|---|---|---|
| . | | | | *Priestia aryabhattai* | NR_115953.1 | 3 |
| . | | | | *Priestia endophytica* | NR_025122.1 | 15 |
| | | | Unknown | *Exiguobacterium sibiricum* | NR_075006.1 | 1 |
| . | | | Caryophanaceae | *Planococcus glaciei* | NR_044384.1 | 1 |
| . | | | | *Planococcus kocurii* | NR_113815.1 | 1 |
| . | | | | *Solibacillus silvestris* | NR_028865.1 | 1 |
| . | | | | *Sporosarcina psychrophila* | NR_113752.1 | 1 |
| . | | | Staphylococcaceae | *Staphylococcus succinus subsp. succinus* | NR_028667.1 | 2 |
| Proteobacteria | γ-proteobacteria | Enterobacteriaceae | Erwiniaceae | *Erwinia persicina* | NR_114078.1 | 3 |
| . | | Lysobacterales | Lysobacteraceae | *Stenotrophomonas chelatiphaga* | NR_116366.1 | 1 |
| . | | | | *Stenotrophomonas rhizophila* | NR_121739.1 | 1 |
| . | | Moraxellales | Moraxellaceae | *Psychrobacter alimentarius* | NR_025798.1 | 1 |
| | | Pseudomonadales | Pseudomonadaceae | *Pseudomonas asturiensis* | NR_108461.1 | 5 |
| | | | | *Pseudomonas azotoformans* | NR_113600.1 | 4 |
| | | | | *Pseudomonas baetica* | NR_116899.1 | 2 |
| | | | | *Pseudomonas brassicacearum subsp. neoaurantiaca* | NR_116299.1 | 3 |
| | | | | *Pseudomonas canadensis* | NR_156852.1 | 6 |
| | | | | *Pseudomonas cedrina* | NR_024912.1 | 6 |
| | | | | *Pseudomonas fluorescens* | LR134318.1 | 2 |
| | | | | *Pseudomonas frederiksbergensis* | NR_117177.1 | 6 |
| | | | | *Pseudomonas kairouanensis* | PP177503.1 | 2 |
| | | | | *Pseudomonas kilonensis* | NR_028929.1 | 7 |
| | | | | *Pseudomonas migulae* | NR_114223.1 | 1 |
| | | | | *Pseudomonas orientalis* | CP027724.1 | 29 |
| | | | | *Pseudomonas poae* | NR_028986.1 | 2 |
| | | | | *Pseudomonas punonensis* | NR_109583.1 | 7 |
| | | | | *Pseudomonas reidholzensis* | NR_157777.1 | 1 |
| | | | | *Pseudomonas trivialis* | NR_028987.1 | 1 |
| | | | | *Pseudomonas viridiflava* | NR_117825.1 | 1 |
